# Shrimp AHPND-causing plasmids encoding the PirAB toxins as mediated by *pirAB*-Tn903 are prevalent in various *Vibrio* species

**DOI:** 10.1038/srep42177

**Published:** 2017-02-07

**Authors:** Jinzhou Xiao, Liyuan Liu, Yiyun Ke, Xiefei Li, Yunfei Liu, Yingjie Pan, Shuling Yan, Yongjie Wang

**Affiliations:** 1 College of Food Science and Technology, Shanghai Ocean University, Shanghai, China; 2 Laboratory of Quality and Safety Risk Assessment for Aquatic Products on Storage and Preservation (Shanghai), Ministry of Agriculture, China; 3 Institute of Biochemistry and Molecular Cell Biology, University of Göttingen, Göttingen, Germany

## Abstract

Acute hepatopancreatic necrosis disease (AHPND) is a newly emerging shrimp disease caused by *pirAB* toxins encoded by a plasmid found in *Vibrio parahaemolyticus*. The *pirAB* toxins are the homologs of the *Photorhabdus* insect-related (Pir) toxins. Here, we report the complete sequences of the AHPND-causing plasmid isolated from *V. owensii*, as well as those of its 11 siblings (pVH family). In addition, we also included 13 related plasmids (pVH-r family) without the *pirAB* genes isolated from a variety of species within the *Vibrio* Harveyi clade. Furthermore, the *pirAB*-Tn903 composite transposon was identified in pVH, and both ends of the transposon appeared to have inserted simultaneously into the ancestor plasmid at different sites. The homologue counterparts of *pirAB* were also detected in a non-pVH plasmid in *V. campbellii*. Taken together, our results provide novel insights into the acquisition and evolution of *pirAB* as well as related plasmids in the *Vibrio* Harveyi clade.

Acute hepatopancreatic necrosis disease (AHPND), originally known as early mortality syndrome (EMS), is a newly emerging disease in shrimp. Since its first outbreak in 2009, the disease has caused serious global economic losses in the shrimp farming industry^1^. It has been confirmed that the bacterium *Vibrio parahaemolyticus*, which contains a 70-kbp plasmid, is the etiological agent of AHPND^1^,^2^,^3^. More specifically, the homologues of the *Photorhabdus* insect-related (Pir) toxins encoded by this plasmid are directly responsible for shrimp mortality in AHPND^1^,^4^.

Interestingly, we have isolated a strain of *V. owensii*, which has been demonstrated as another causative agent of AHPND, in Shanghai, China in our previous study^5^. Meanwhile, Kondo *et al*. also reported an AHPND-causing bacterial strain *V. harveyi* in Northern Vietnam^6^. Importantly, these two non-*V. parahaemolyticus* AHPND-causing strains were found to contain plasmids carrying the genes for homologues of the Pir toxins produced by *V. parahaemolyticus*. Furthermore, a number of other *Vibrio* strains have been shown to be involved in causing AHPND in shrimp^6^,^7^,^8^,^9^,^10^. However, it remains unclear whether these strains also contain similar AHPND-causing plasmids that might be related to each other.

In order to identify, as well as better understand the evolutionary relationship between toxin genes-encoding plasmids and the corresponding toxin genes, we subjected the AHPND-causing plasmids, the toxin genes and the related whole genome sequences available in GenBank to comprehensive analysis using a variety of bioinformatic approaches. Our results provide novel insights into the diversity, acquisition and evolution of the toxin genes and their related plasmids in the *Vibrio* Harveyi clade.

## Materials and Methods

### Isolation of the *V. owensii* SH-14 strain with the natural deletion of the *pirAB* genes (*pirAB* ^−^)

A single colony was picked and re-suspended in 50 ml fresh TSB15 culture medium (tryptic soy broth supplemented with 1.5% NaCl) after overnight culture of the *V. owensii* SH-14 isolate (*pirAB*^+^)^5^ on TSA15 plate (tryptic soy agar supplemented with 1.5% NaCl). The bacteria were incubated at 30 °C for 12 hours with 220 rpm shaking. One ml of the 12 hour-culture was transferred to 50 ml of fresh TSB15 medium, which was cultured as described above. The transfer was repeated for five times.

One hundred microliter of diluted bacterial cells (10^−8^ to 10^−9^) from each generation (transfer culture) was spread on TSA plate. Single colony was screened for the lack of *pirAB* by using PCR with AP1-F16/AP2-R1 primer set^1^, and the DNA sequences of PCR products were determined to further confirm the natural deletion of *pirAB*.

### Immersion-challenging bioassay

Both the *pirAB*^+^ and the *pirAB*^−^ strains of *V. owensii* SH-14 were used to challenge shrimp *Litopenaeus vannamei.* Thirty SPF shrimp (0.5–2 g) were maintained in tanks containing 19.8 L artificial seawater (13‰ salinity, 27 °C) for 3 days. Two hundred ml of bacterial culture at bacterial density of approximately 10^8^ CFU/ml (OD600 = 0.6–0.8) was added directly to the experimental tanks to obtain an approximate bacterial density of 10^6^ CFU/ml. Shrimp in the negative control group were immersed in sterile TSB15. Three replications were applied to each treatment.

### SDS-PAGE and Western Blot

The *pirAB*^+^ strain and *pirAB*^−^ strain of *V. owensii* SH-14 were cultured separately in TSB15 at 30 °C for 12 hours with 220 rpm shaking. Then, 50 ml of the culture was collected, centrifuged at 6,780 × g for 20 min at 4 °C to remove the cell debris. The crude proteins in supernatant were precipitated by ammonium sulfate (AS) referring to the method described by Sirikharin and coauthors^4^ with slight modifications. Briefly, AS was added into the supernatants by batches until its concentration reached 40% of saturation (e.g. 11.3 g for 50 ml supernatant) at 0 °C. Proteins were allowed to precipitate for 30 min at 0 °C before centrifugation at 13,500 × g for 20 min at 4 °C to collect pellets. The AS precipitated fraction was re-suspended by adding pre-cooled ddH_2_O and kept at −80 °C before use.

After SDS-PAGE, the separated proteins were transferred to PVDF membrane (150 mA, 55 min) and blocked overnight with 5% (wt/vol) non-fat milk in 10 × TBS buffer (40 g NaCl, 1 g KCl, 15 g Tris in 500 mL ddH_2_O, pH 7.4) at 4 °C. Subsequently, it was sequentially incubated with the anti-PirAB antibodies^1^ (rabbit) and the secondary antibody conjugated with AP (alkaline phosphatase) (goat anti rabbit) for 1 h at room temperature. NBT/BICP was used as the chromogenic substrate, and the chemical color reaction was incubated approximately for 5 min.

### NGS of the bacterial genome of *Vibrio owensii* SH14 and the plasmid pVH_vo_

The genomic DNA of *Vibrio owensii* SH14 was extracted with a TIANamp Bacteria DNA Kit (TIANGEN, Beijing). The extracted DNA samples were sequenced on an Illumina MiSeq sequencer and assembled^11^ into 120 scaffolds (Majorbio Bio-Pharm Technology Co., Ltd., Shanghai, China) as reported previously^5^.

The contigs were mapped using pVPA3-1 (KM067908) as a reference in order to assemble the scaffolds of plasmid pVH_vo_ using Geneious Pro (version 6.1.2; Biomatters Ltd.). Primer sets were designed, and amplicons were sequenced by the Sanger method to fill the gaps and to confirm overlaps between contigs. The prediction and annotation of open reading frames (ORFs) on pVH_vo_ were conducted according to the procedures described previously^12^,^13^. Each predicted ORF encompassed a start codon of ATG, had a minimum size of 120 bp, exhibited standard genetic code, and contained a stop codon. BlastP program (http://blast.ncbi.nlm.nih.gov/) was used for sequence similarity comparisons of the predicted ORFs to NCBI non-redundant protein sequences (nr) database.

### Identification and assembly of pVH plasmids in WGS datasets

To identify pVH plasmids in public dataset, we first established a database of all of the complete AHPND-causing plasmids, i.e. pVA1 (KP324996), pVPA3-1 (KM067908) and pVH_vo_ (KX268305). Then, AHPND toxin genes *pirA* and *pirB* on pVH_vo_ were searched (megablast^14^) respectively against NCBI WGS database with search results limited to “*Vibrio*”. WGSs containing *pirA* or *pirB* gene were downloaded and compared to the AHPND-causing plasmid local database mentioned above using local blast^15^ (blastn E-value < 10^−1^). Contigs matching any of the sequences in this local database were assembled respectively using Geneious Pro with the sequence of pVA1 as reference (Highest sensitivity, Fine Tuning: iterate up to 5 times) (version 6.1.2; Biomatters Ltd.). The obtained sequences were checked and corrected manually as needed.

### Identification of *pirAB*-Tn903

To identify transposons on pVH_vo_, plasmid dot-plot (self) was generated with Geneious Pro (version 6.1.2; Biomatters Ltd.) and checked manually. Any inverted sequences flanking the *pirAB* genes or the transposase ORFs were recorded for further analysis. PSI-Blast program^16^ was used to search for homologues of transposase encoded in pVH_vo_ within the NCBI non-redundant protein sequences (nr) database. Annotation of transposon was based on its length, inverted repeats and transposase.

To verify the location of *pirAB*-Tn903 within different pVH plasmids assembled in this study, the consistency of sequence identity of the *pirAB* containing contigs, the ORF arrangement of its adjacent contigs and the location of gaps between the ORFs were checked manually.

### Identification of pVH-r contigs and complete sequences

To identify pVH-r contigs and complete sequences in public datasets, the sequence of *pndA* in pVH_vo_ was searched (megablast^14^) against NCBI nr database and WGS database as limited by the different taxonomic ranks. Sequences that matched *pndA* were downloaded. Their relationship to pVH plasmid was identified based on alignment with sequence of pVH_vo_ using Mauve^17^.

### Identification of pVo-VH plasmid

A mock sequence containing ORF20 and 29 in pVH_vo_ was designed. Nucleotide sequence between these two ORFs in pVH_vo_ was replaced with the same amount of “N” nucleotides. The mock sequence was then searched (megablast^14^) against NCBI WGS database with search results limited to “*Vibrio*”. Contigs with hits were downloaded and checked manually based on dot-plot analysis with the sequence of pVH_vo_. Other contigs of pVo-VH were investigated in the same WGS using local blast^15^ (blastn, e-value = 0) with the sequence of pVH_vo_ as query.

### Coverage and sequence identity analyses

Sequence identity and coverage of the plasmids pVH, pVH-r and pVo-VH versus pVH_vo_ were compared using megablast algorithm with default parameters. Plots were generated using BRIG 0.95^18^ with 70% and 50% as the upper and lower identity threshold, respectively.

### Phylogenetic analysis

The amino acid sequence of PirB or IS903 transposase was compared to sequences in public databases using PSI-BLAST^16^. Related sequences were retrieved. Only those sequences with identity of more than 25% and coverage of more than 85% were incorporated into this study. Amino acid sequences were aligned using MUSCLE^19^, and phylogenetic analysis was conducted using MEGA 6^20^ with maximum-likelihood algorithm.

### Nucleotide sequence accession numbers

The sequence of plasmid pVH_vo_ was deposited into the GenBank database (accession no. KX268305).

## Results and Discussion

### The *pirAB* genes in *V. owensii* SH-14 are responsible for shrimp AHPND

A total of 465 colonies were subjected to screening by using PCR detection, which resulted in 18 colonies with the natural deletion of the *pirAB* genes (4% excision rate). One of these 18 colonies, named the *pirAB*^−^ *V. owensii* SH-14 strain, was further verified via Sanger sequencing (Fig. S1) and used in the subsequent bioassay and Western blot test.

**Figure 1:**
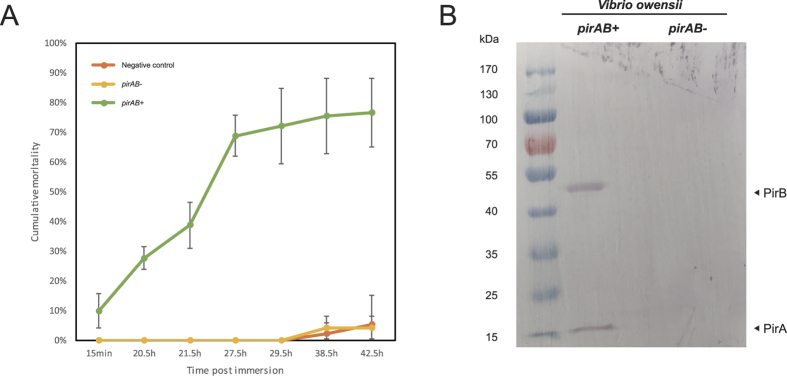
Toxicity analysis of the *pirAB* genes in *V. owensii*. (**A**) Shrimp mortality after challenged with either the *pirAB*^+^ or *pirAB*^−^ *V. owensii* strains. Error bar represents the standard deviation (SD) values between three groups of parallel experiment; (**B**) Western blot analysis of the expression of PirAB toxin proteins in two *V. owensii* strains.

Immersion-challenging bioassay showed that the *pirAB*^+^ *V. owensii* SH-14 strain caused severe AHPND in shrimp (up to 90% of mortality). In contrast, the *pirAB*^−^ *V. owensii* SH-14 strain revealed no difference in mortality (up to 5%) in comparison to the negative control (Fig. 1A). In addition, based on Western blot assay, the PirA and PirB toxin proteins were detected in the *pirAB*^+^ *V. owensii* SH-14 strain but absent in the *pirAB*^−^ *V. owensii* SH-14 strain (Fig. 1B). Taken together, these results clearly indicate, like in *V. parahaemolyticus*^1^, the *pirAB* genes in *V. owensii*^5^ are also the toxic ones and responsible for the mortality of the infected shrimp (Fig. 1).

### Shrimp AHPND-causing plasmids were identified in various *Vibrio* species of the Harveyi clade

**Figure 2:**
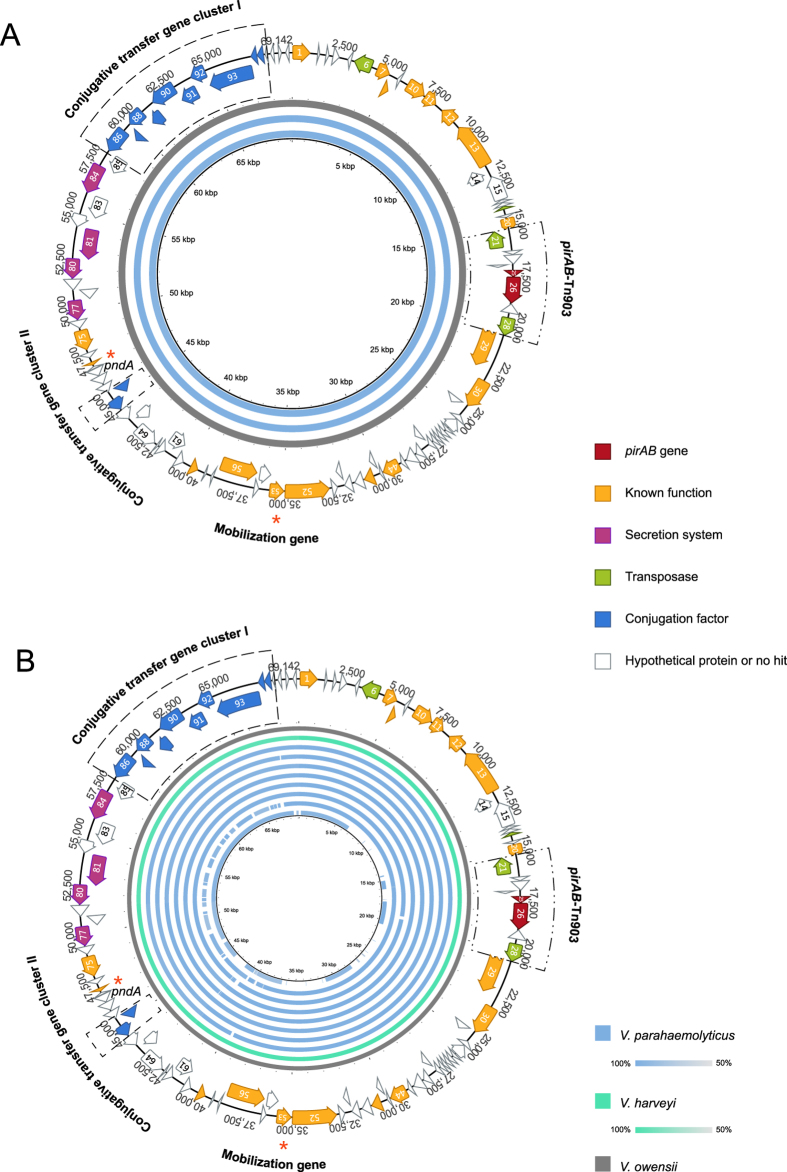
Sequence map of the AHPND-associated plasmid pVH_vo_ in *V. owensii* and sequence identity plot of pVH plasmids. All predicted open reading frames (ORFs) are shown as arrows in different colors. Direction of arrowhead represents the transcriptional orientation. The *pndA* and mobilization genes are labeled with red asterisks. (**A**) Sequence identity plot (>99%) of pVH_vo_ (gray), pVPA3-1 (blue) and pVA1 (blue, inner circle). (**B**) Sequence identity plot (>99%) of pVH_vo_ (gray), pVH_vh_1 (green), pVH_vp_1-8 (blue, outer to inner). The sequence of pVH_vo_ was used as reference.

The complete sequence of AHPND-causing plasmid, named pVH_vo_ (accession no. KX268305), in *Vibrio owensii* strain SH-14, was obtained using MiSeq and Sanger sequencing. It is 69,142 bp in size with 99 predicted open reading frames (ORFs) (Fig. 2 and Table S1). The sequence of pVH_vo_ revealed 99% identical to the AHPND-causing plasmids of pVPA3-1 (69,168 bp, KM067908) and pVA1 (70,452 bp, KP324996), which were detected in *V. parahaemolyticus*^1^,^3^ (Fig. 2A and Table S2). Accordingly, these three plasmids were assigned to the same plasmid family tentatively named pVH.

Based on results from data mining, eight datasets of WGSs available in GenBank were found to contain the toxin genes of *pirAB* (Table S3). The sequences of the contigs (3.1 to 3.5 kb) that contain the *pirAB* genes were almost identical (>99%) to each other (Fig. 2B). Subsequently, the sequence of pVA1 (KP324996) was used as the reference template to assemble the corresponding plasmids in each of these eight WGSs. The resulting eight potential plasmids contained nearly complete sequences (small gaps exist between some un-overlapped contigs) of 62 to 70 kbp that share 99% of sequence identity with pVH_vo_ (Fig. 2B and Table S3). Each of the sequence of *pirA* and *B* is identical across these 12 plasmids with the only exception of pVH_vp_7. It had one more base in *pirB*, which likely resulted from the error rate in NGS.

In addition, *pirB*, but not *pirA*, was detected in another WGS (JPLV01000000), but it only contains partial sequence (89%) of *pirB* and aligns to 57% of the sequence length of pVH_vo_ with over 95% identity (Fig. 2B and Table S3). This result could possibly be attributed to variable sequencing quality and depth^21^. In fact, there was no significant difference between this potential plasmid (pVH_vp_8) and the others analyzed.

These nine newly discovered plasmids were named pVH_vh_1 and pVH_vp_1-8, respectively (Table S3). They were assigned to the same pVH family because they are almost identical to the sequences of pVH_vo_, pVPA3-1 and pVA1.

Currently, the pVH plasmid hosts include all known shrimp AHPND-causing *Vibrio* spp. (Tables S2 and S3). These pathogens are more closely related to each other than to other *Vibrio* species, and all of them belong to the *Vibrio* Harveyi clade in the family of *Vibrionaceae*^22^,^23^.

### The shrimp AHPND-causing plasmids contain Tn903-like composite transposon

Identical transposons were identified upstream and downstream of the toxin genes (*pirAB*)-containing region of pVH (Fig. 2 and Fig. S2A). It was characterized as a transposase gene (*tnp*), and is 1,054 bp in size with 18 bp of terminal inverted repeats (IRs) (Fig. S2A), which are characteristics similar to those of IS903 in *Escherichia coli*^24^. However, compared with the *E. coli* IS903, three point mutations, indicated with parenthesis, were observed in the IRs of 5′GA(A)TTA(CG)CAACAAAGCC3′ of the *Vibrio* IS903^24^. Moreover, the *tnp* of the *Vibrio* IS903 shares 60% of amino acid identity with its homologue counterpart in *E. coil* (plasmid, accession no. CDF31572.1). However, there was no significant similarity between the two on the nucleic acid level. In contrast, the *Vibrio* IS903 *tnp* exhibits 56–97% similarity to its homologues in *Shewanella*. Taken together, our results indicate an ancient divergence of IS903 in *Vibrio* and *E. coli*.

The *Vibrio* IS903 that was detected on both ends of the *pirAB* containing region was characterized by an inverted orientation (Fig. S2A), which is one of the classical features of composite transposon^25^. Accordingly, the IS903-*pirAB*-IS903 sequence was classified as the *pirAB*-Tn903 composite transposon (Fig. S2).

### Both ends of the *Vibrio* Tn903-like composite transposon inserted simultaneously into the ancestral plasmid of pVH at different target sites

Unlike the classical transposons discussed above (Fig. S2B-1 and B-2), direct repeats flanking the insertion sites of the *Vibrio* Tn903-like composite transposon were not detected, although a 9 bp of direct repeat (GTTTGTTTC) was found to flank an independent IS903-like transposon in pVA1 (9,145–10,198 bp) (Fig. S2C). This observation confirms the duplicative feature of the insert sites of IS903 upon transposition in *Vibrio*.

**Figure 3:**
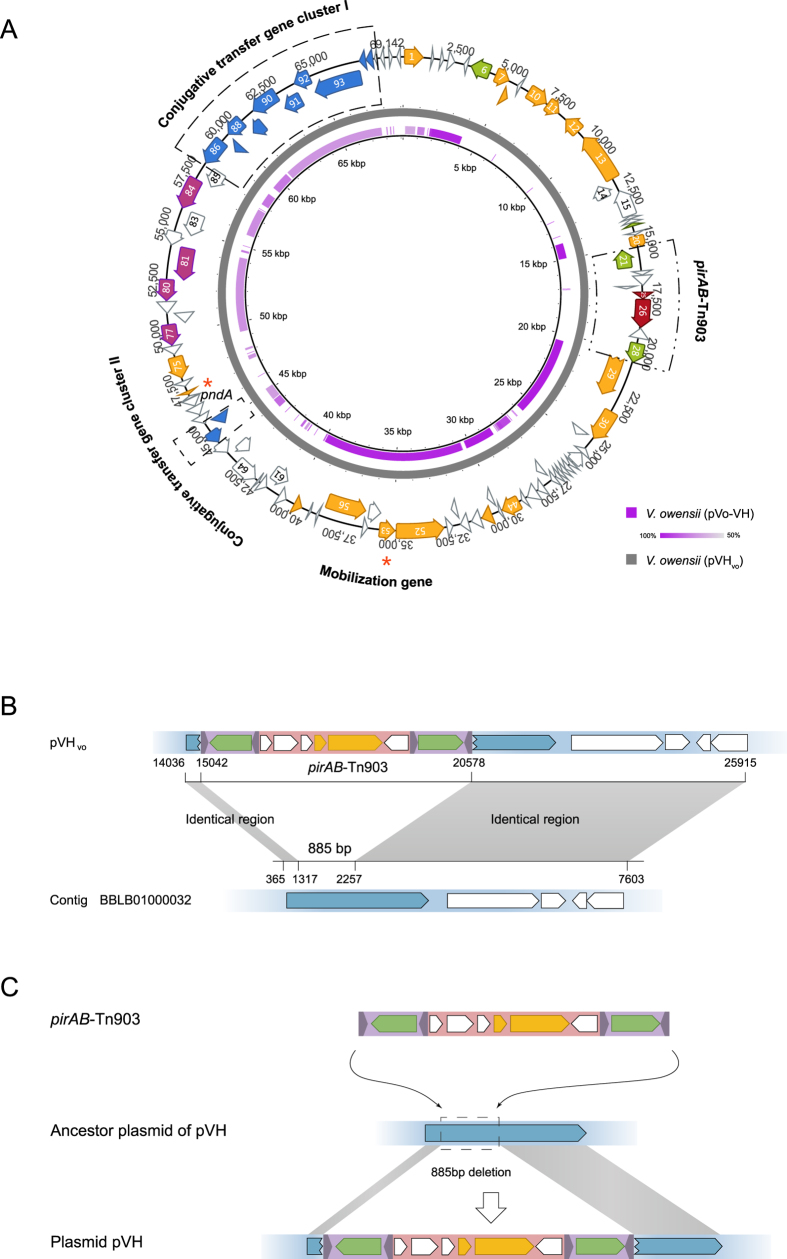
Sequence comparison between pVH_vo_ and pVo-VH, and *pirAB*-Tn903 transposition. (**A**) Sequence identity plot based on comparing pVo-VH with pVH_vo_. The outer map is the reference sequence map of pVH_vo_. Color intensity is proportional to sequence identity. (**B**) Sequence comparison of pVH_vo_ with contig BBLB01000032 of pVo-VH. Identical regions between them are indicated in gray. Putative ORFs of pVH_vo_ and pVo-VH are shown as arrowed boxes. (**C**) *pirAB*-Tn903 transposition. The *pirAB*-Tn903 inserted into ancestor of pVH at two target sites, which resulted in the deletion of an 885 bp of sequence on the plasmid ancestor.

Interestingly, a *V. owensii* assembly (BBLB01000000) was found to contain at least four contigs of more than 50 kb total, that aligns with the majority of pVH_vo_ (Fig. 3A). One of these contigs (BBLB01000032) revealed more than 99% identical to pVH_vo_ (Fig. 3B). This suggests that the isolated *V. owensii* likely hosted an unknown plasmid, named pVo-VH, which is related to pVH.

Further analysis of the 7.6 kb of this particular contig (BBLB01000032) revealed that the sequences upstream and downstream of an 885 bp fragment (1,372–2,256 bp), which is absent in pVH, shared more than 99% of identity with that of the Tn903-like composite transposon in pVH (Fig. 3B). Both the 5 and 3 prime ends of the ORF, which contains the 885 bp of sequence in the middle, identified within the contig BBLB01000032 are also identical to the ORFs that flanked *pirAB*-Tn903 in pVH (Fig. 3B). Furthermore, all of these ORFs encode proteins in the methylase family, indicating that the homologue counterpart of this ORF was present within the common ancestor of pVH and pVo-VH, and subsequently split into two insertions of *pirAB*-Tn903 in the pVH family (Fig. 3C).

Furthermore, given that the 885 bp sequence that corresponds to the insertion site of Tn903 in the pVo-VH plasmid is absent in pVH (Fig. 3B), it is reasonable to speculate that both ends of Tn903 inserted simultaneously into the different target sites that flanked the 885 bp sequence in the ancestor plasmid of pVH (Fig. 3C and Fig. S2B-3). This could have explained the absence of both the 885 bp sequence, as well as the target repeats, in pVH (Fig. 3C and Fig. S2B-3).

Besides *V. owensii, V. harveyi* and *V. parahaemolyticus*, similar Tn903-like composite transposons were also detected in either the plasmids or the whole genomes of various strains of *V. anguillarum* and *V. campbellii* of the Harveyi clade (LK021128, CP006700, CP006701, CP006606 and CP000790).

### A pVH-related plasmid family is prevalent in various *Vibrio* species of the Harveyi clade

To better understand whether plasmids that are related to pVH exist, the gene *pndA* was used as a hallmark for WGS database search due to its presence in all pVH plasmids described above and its potentially important role in pVH plasmid inheritance^1^.

With database search restricted to *Vibrio* (Genus), 32 contigs showing *pndA*^+^ (84–100% of identity to pVH_vo_) results were detected in 22 different WGSs. No additional contigs were observed when *Vibrio* was changed to *Vibrionaceae* (Family) or *Vibrionales* (Order) during data mining. After removing the 11 WGSs that contain the pVH described above, 11 WGSs that are *pndA*^+^, but *pirAB*^−^, were obtained (Table S4). Each of these 11 WGSs encompasses one *pndA*^+^ contig with a length of 736 to 100,302 bp (Table S4). For the ease of comparison, these 11 contigs were tentatively defined as sequences of a pVH-related plasmid family named pVH-r.

Ten of the pVH-r sequences were found in *V. parahaemolyticus* except for one that was detected in *V. hyugaensis* (Table S4). All of these *Vibrio* isolates are not the causative agents of shrimp AHPND, even though they belong to the Harveyi clade^23^,^26^. This suggests that the pVH-r plasmids are *Vibrio* Harveyi clade specific. This finding is also in agreement with the host specificity of the pVH family described above.

**Figure 4:**
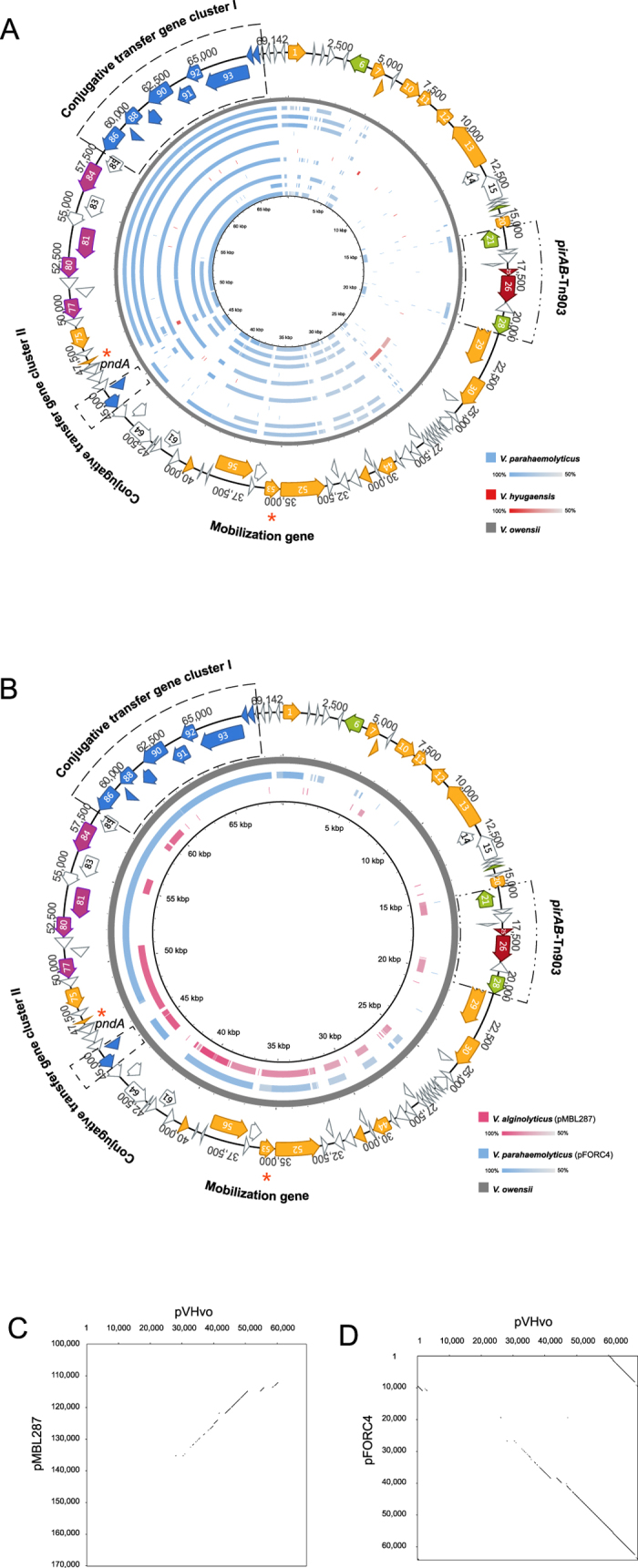
Sequence comparison between pVH and pVH-r. (**A**) Sequence identity plot of pVH-r contigs based on comparison to pVH_vo_. The outer map is the reference sequence map of pVH_vo_. From outer to inner: pVH_vo_ (gray), AMRZ01000017 (blue), AWHI01000014 (blue), AWIH01000044 (blue), AWJX01000468 (blue), AXNR01000072 (blue), BBLF01000195 (red), JPIP01000006 (blue), JPKV01000063 (blue), JPLU01000004 (blue), JPLV01000074 (blue), and LIRR01000030 (blue). (**B**) Sequence identity plot of the two complete pVH-r plasmids based on comparison to pVH_vo_. The outer map is the reference sequence map of pVH_vo_. The color intensity is proportional to sequence identity. (**C**) Dot plot comparison of pVH_vo_ and pMBL287 indicating the regions containing shared identical sequence. (**D**) Dot plot comparison of pVH_vo_ and pFORC4 (partial) indicating the regions containing shared identical sequence.

**Figure 5:**
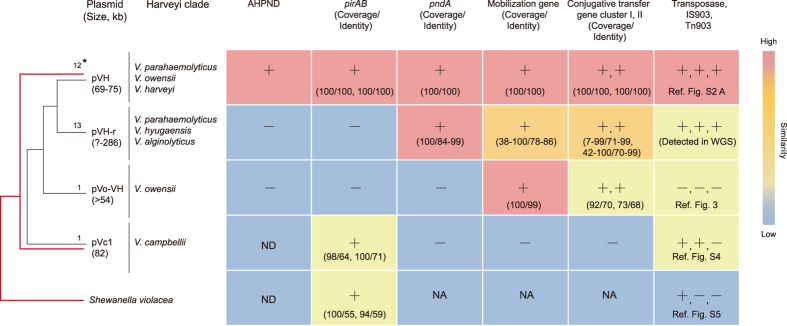
Schematic diagram showing the potential relationships between pVH and the other related plasmids. Red lines indicate the phylogeny of *pirB* in *Vibrio* and *Shewanella*. *The number of complete plasmid sequences or contigs, ^+^Yes or present, ^−^No or absent, ND: Not determined, NA: Not applied.

In addition to *pndA*, the pVH-r sequences also share several conserved regions with pVH as revealed by sequence alignment analysis (Fig. 4A and S3). Most of the pVH-r sequences share conserved regions similar to the two conjugative transfer gene clusters^1^,^3^ presented on pVH with an 89–99% of sequence identity (Fig. 4A and Table S5). As for some short contigs, such as BBLF01000195 and JPKV01000063, similar gene clusters were found in other contigs in their corresponding WGSs with up to 99% of identity to pVH (Fig. 4A and Table S5). Additionally, homologues (78–86% of identity) to the mobilization protein gene (ORF53 on pVH_vo_) on pVH were also detected in all of these eleven *pndA*^+^ *pirAB*^*—*^ WGSs (Figs 4A and 5 and Table S5).

Two plasmids, pFORC4 in *V. parahaemolyticus* (64,049 bp, CP009849) and pMBL287 in *V. alginolyticus* (286,750 bp, CP013487) of the Harveyi clade, are also related to pVH-r (Fig. 4B, Table S6). They contain *pndA*, the conjugative transfer gene clusters, the mobilization protein gene and the IS903-like transposon (absent in pFORC4), but not the *pirAB* toxin genes (Figs 4B and 5 and Table S6).

Notably, except for the conserved regions, large fragments that had no significant similarity with pVH were detected in the pVH-r sequences (Fig. 4 and S3). This suggests that pVH and pVH-r diverged early on from a common ancestor (Fig. 5).

### Homologue counterparts of *pirB* in *V. parahaemolyticus, V. harveyi* and *V. owensii* were detected in a non-pVH plasmid in *V. campbellii*

**Figure 6:**
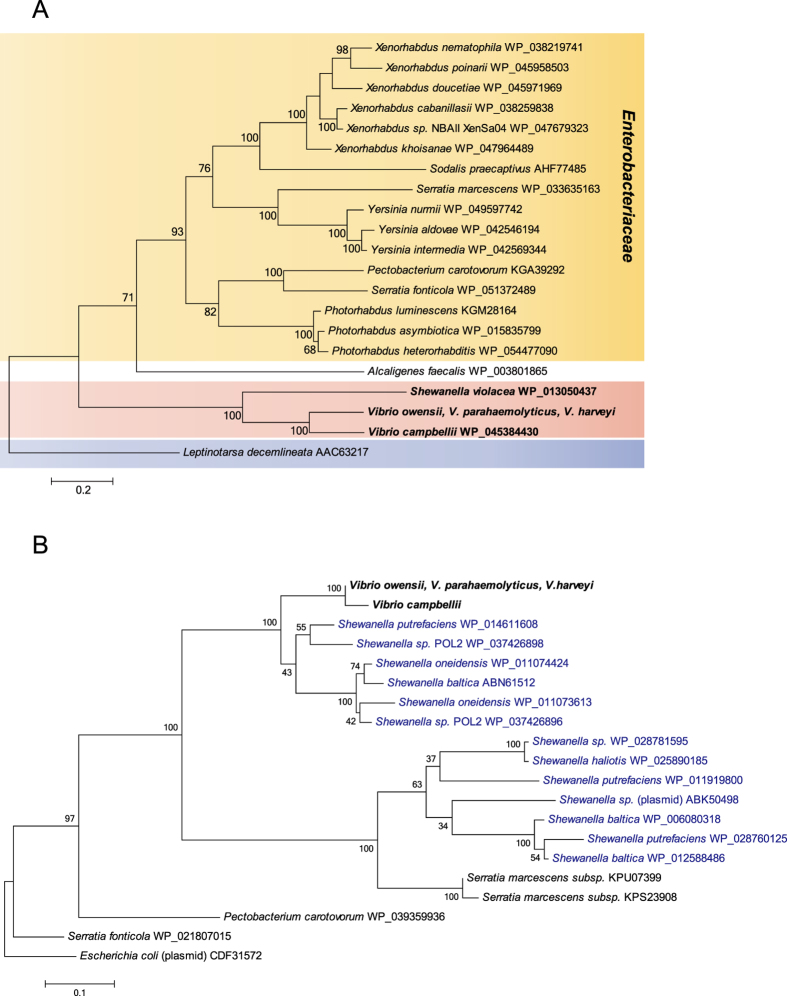
(**A**) Phylogeny of PirB in *Vibrio* and its homologs. The Maximum Likelihood tree was rooted using the juvenile hormone esterase-related protein of *Leptinotarsa decemlineata* as outgroup. *Vibrio* and *Shewanella* are indicated in bold. Multiple sequence alignments were performed with MUSCLE. The robustness of the tree was evaluated using bootstrap analysis (1,000). Percentage values are indicated at the nodes. Bar = 20 substitutions per 100 amino acids. (**B**) Phylogeny of the IS903 transposase encoded by pVH plasmids and its homologues. The Maximum Likelihood tree was rooted using *E. coli* IS903 transposase as outgroup. *Vibrio* is indicated in bold, and *Shewanella* in blue. Multiple sequence alignments were performed by using MUSCLE. The robustness of the tree was evaluated using bootstrap analysis (1,000). Percentage values are indicated at the nodes. Bar = 10 substitutions per 100 amino acids.

To understand the phylogenetic affiliation of the shrimp AHPND-causing toxin gene, homologues of the PirB protein were investigated using PSI-Blast and then included in phylogenetic analysis. As shown in Fig. 6A, PirB of the pVH family (100% of identity) in *V. parahaemolyticus, V. harveyi* and *V. owensii* belongs to the same monophyletic group as their homologue in *V. campbellii* (70% of identity), which is also a member of the *Vibrio* Harveyi clade. Thus far, the PirB in *V. campbellii* represents the closest homologue to that identified in pVH. In addition, PirB in *Vibrio* shares a common ancestor with that in *Shewanella violacea* (44% of identity). Interestingly, distantly related PirB homologues, which belong to the same group, were widely detected in different genera of the *Enterobacteriaceae* family. Most of these enterobacterial species are pathogens in insects. Notably, the juvenile hormone esterase-related protein of *Leptinotarsa decemlineata* (28% identity) was found to be homologous to PirB, an observation consistent with that reported by Waterfield *et al*.^27^.

Surprisingly, the homologue counterpart of *pirB* in *V. campbellii* was located within a non-pVH extrachromosomal element named pVc1 (82,408 bp, NZ_BBKW01000191) (Fig. S4). The pVc1 element was determined as non-pVH based on the following reasons: (I) 35% of the sequence of pVc1 share 95% of sequence identity with the plasmid pVIBHAR (89,008 bp, NC_009777) of *V. campbellii* ATCC BAA-1116, (II) the ORFs neighboring the homologues of *pirAB* genes on pVc1 exhibit no significant similarity to that in pVH, and (III) the conserved gene of *pndA* in pVH and pVH-r families is absent in pVc1. Moreover, homologues of the conjugative transfer gene clusters and the mobilization gene on pVH are also absent in pVc1 (Fig. S4 and Fig. 5). Taken together, these observations suggest that *pirB* in *Vibrio* likely had undergone independent extrachromosome (plasmid)-driven evolution.

An IS903-like (ORF58, 84.3% of nucleotide identity) and a truncated IS903-like (ORF8, 76.9% of identity) transposons were identified upstream and downstream of *pirAB* in pVc1, respectively (Fig. S4). Interestingly, the transposase genes on these transposons are inserted in the same orientation and are 54 kbp apart. It is unclear whether pVc1, like pVH, acquired the *pirAB* gene through a Tn903-mediated mechanism.

The pVc1 IS903 *tnp* in *V. campbellii* was phylogenetically categorized with the pVH IS903 *tnp* in *V. parahaemolyticus, V. harveyi* and *V. owensii* (Fig. 6B). Interestingly, similar to the *pirB* gene, the IS903 *tnp* appears to derive from a common ancestor as the *tnp* in *Shewanella.* Furthermore, the *Vibrio* IS903 *tnp* seems to have originated from *Shewanella* (Fig. 6B). Although the *tnp* genes were coincidently detected to flank the *pirAB* in *S. volacea* (Fig. S5), they share no significant similarity with neither the *Vibrio* IS903 *tnp* nor with each other. In addition, no IR associated with transposon was detected in the *tnp* of *S. volacea*.

In conclusion, the AHPND-causing pVH plasmids were identified in three different species of *Vibrio* isolated from different geographical locations at different time (Tables S2 and S3), and a pVH-related plasmid family is prevalent in various *Vibrio* species of the Harveyi clade. The *E. coli* Tn903-like composite transposons are widely detected in a variety of *Vibrio* species of the Harveyi clade (Fig. 5). In contrast to the classical transposons, both ends of the *Vibrio pirAB*-Tn903 composite transposon appeared to have inserted simultaneously into the ancestor plasmid of the AHPND-causing plasmids of the pVH family at different target sites. Taken together, these findings provide novel insights into the essential role played by the pVH plasmids in the global outbreaks of shrimp AHPND.

## Additional Information

**How to cite this article**: Xiao, J. *et al*. Shrimp AHPND-causing plasmids encoding the PirAB toxins as mediated by *pirAB*-Tn903 are prevalent in various *Vibrio* species. *Sci. Rep.* **7**, 42177; doi: 10.1038/srep42177 (2017).

**Publisher's note:** Springer Nature remains neutral with regard to jurisdictional claims in published maps and institutional affiliations.

## Supplementary Material

Supplementary Information

Supplementary Dataset 1
