# Supplementary material for: Shrimp AHPND-causing plasmids encoding the PirAB toxins as mediated by pirAB-Tn903 are prevalent in various Vibrio species
Source: Sci Rep. 2017 Feb 7;7:42177. doi: 10.1038/srep42177 (PMC5294582; doi:10.1038/srep42177)
Supplement: Supplementary Information [file srep42177-s1.pdf]

Supplementary information

Shrimp AHPND-causing plasmids encoding the PirAB toxins as mediated by *pirAB*-Tn903 are prevalent in various *Vibrio* species

Jinzhou Xiao[a], Liyuan Liu[a], Yiyun Ke[a], Xiefei Li[a], Yunfei Liu[a],

Yingjie Pan[a,b], Shuling Yan[a,c], Yongjie Wang[a,b]

[a] College of Food Science and Technology, Shanghai Ocean University, Shanghai, China;

[b] Laboratory of Quality and Safety Risk Assessment for Aquatic Products on Storage & Preservation (Shanghai), Ministry of Agriculture, China;

[c] Institute of Biochemistry and Molecular Cell Biology, University of Goettingen, Goettingen, Germany

Corresponding author: Yongjie Wang

Tel.: +86 21 61900505; Email: yjwang@shou.edu.cn (Y. J. Wang).

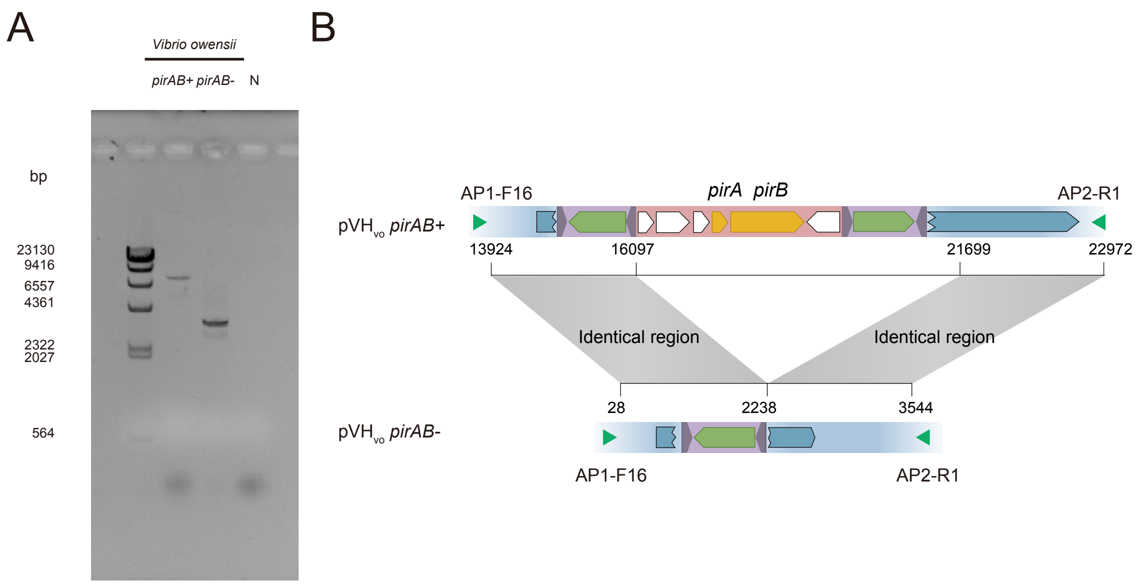

Fig. S1 The natural deletion of the *pirAB* genes in *V. owensii*.

(A) PCR analysis of the deletion of the *pirAB* in *V. owensii*; (B) Schematic presentation of the deletion of the *pirAB* in the associated plasmid in *V. owensii*.

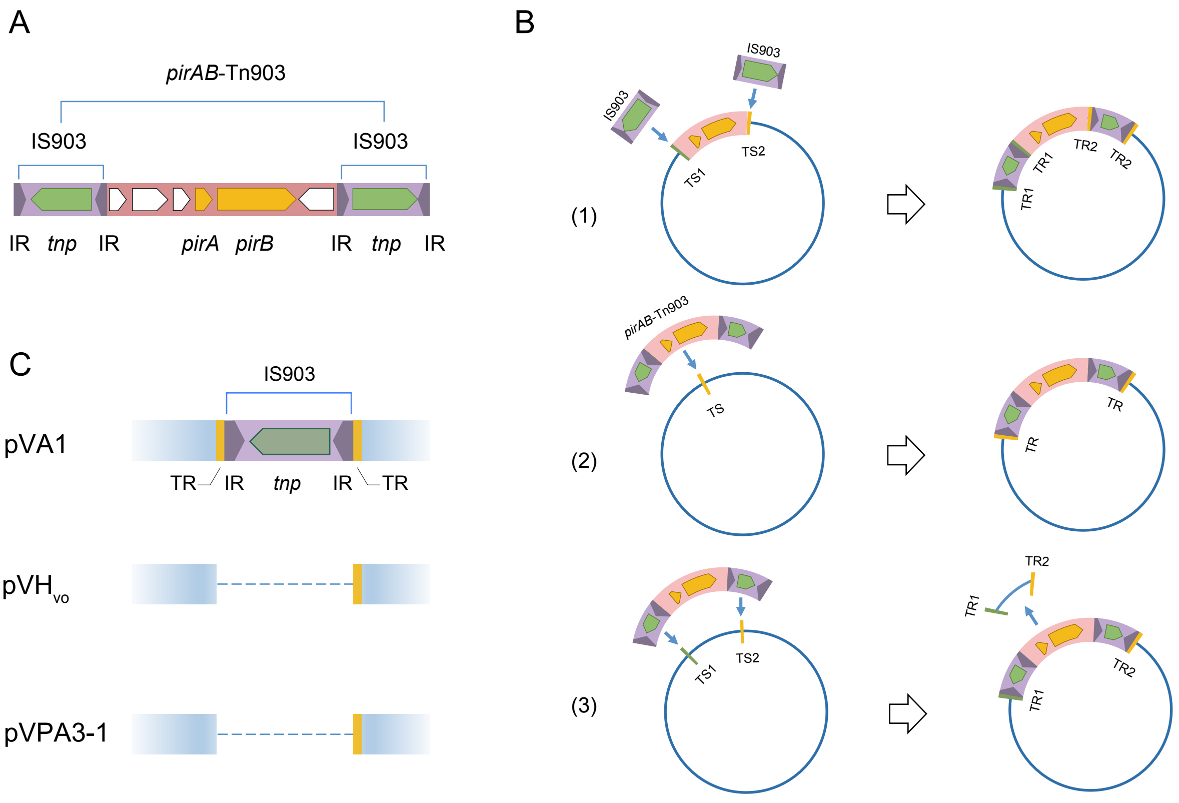

Fig. S2 (A) Schematic diagram of *pirAB*-Tn903 composite transposon. Composite transposon Tn903 contains a central region that harbors the *pirAB* toxin genes flanked by IS903 elements. The IS903 elements contain the 18 bp inverted terminal repeats (IRs) and the transposase gene (*tnp*). The two IS903 elements are identical and are in inverted orientation. **(B) Three potential transposition mechanisms of *pirAB*-Tn903**. (B1) Two independent IS903 transposons inserted into the ancestor of pVH_{vo} at two target sites (TS), respectively. In this scenario, the two transposons would be flanked by different target repeats (TR). (B2) *pirAB*-Tn903 inserted into the ancestor of pVH_{vo} at a single target site. In this scenario, the whole *pirAB*-Tn903 transposon would be flanked by the same target repeats. (B3) *pirAB*-Tn903 inserted into the ancestor of pVH_{vo} at two target sites. In this scenario, the sequence between these two target sites would be replaced by *pirAB*-Tn903, and neither ends of the IS903 elements nor the *pirAB*-Tn903 composite transposon would contain the target repeats. **(C) IS903 transposon in pVA1**. Insertion of an independent IS903 transposon that was absent in pVH_{vo} and pVPA3-1 generates the 9 bp of target repeats (TR) on pVA1. IR: 18 bp of terminal inverted repeat; *tnp*: transposase gene.

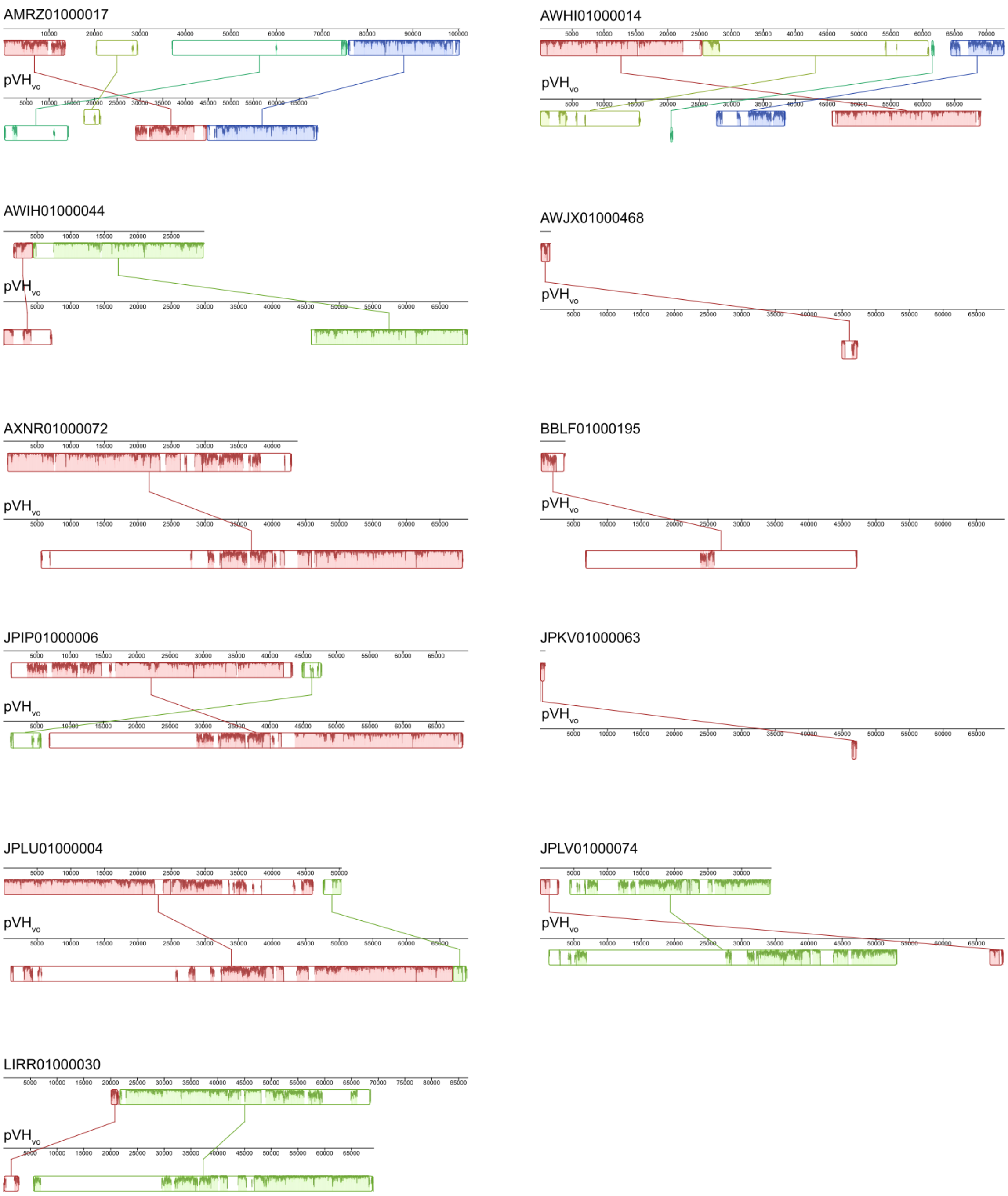

Fig. S3 Sequence alignments of pVH_{vo} and pVH-related contigs. Sequence inversion and rearrangement of conserved regions are indicated in different colors.

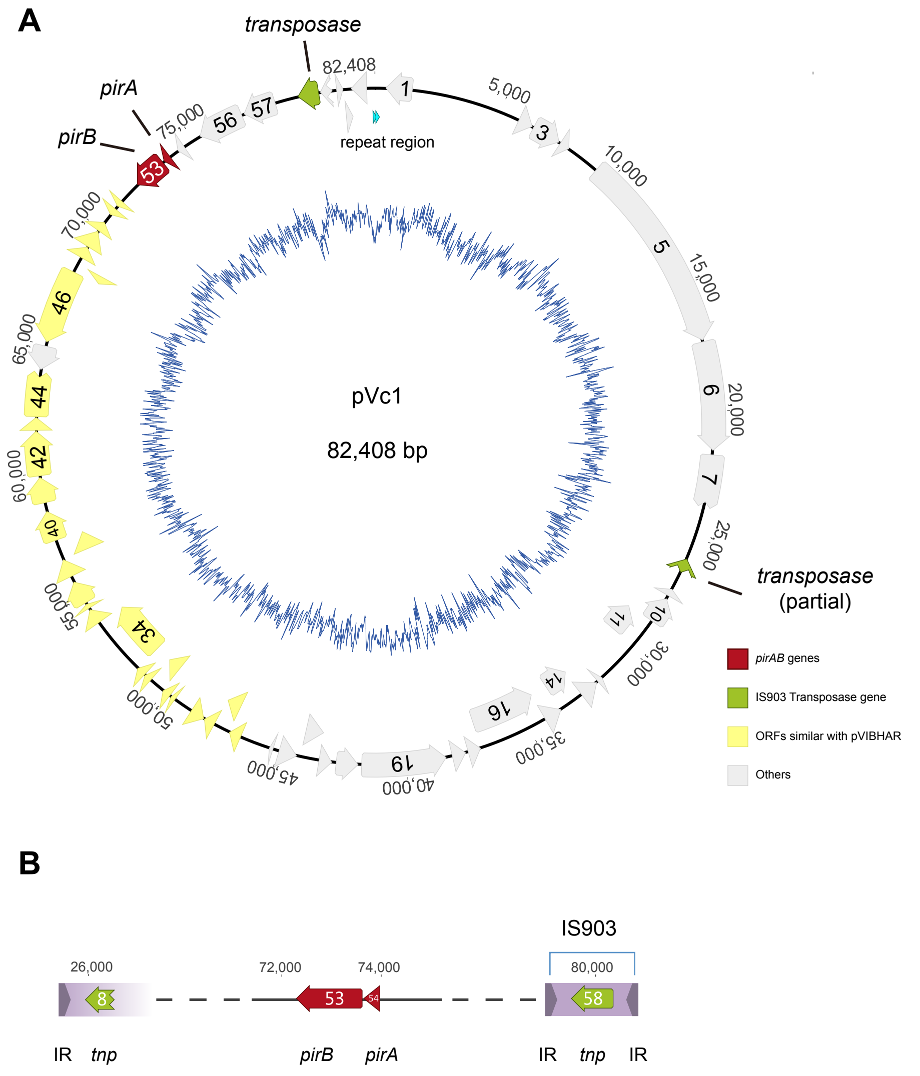

Fig. S4 Sequence map of the putative pVc1 plasmid in *V. campbellii*. (A) All predicted open reading frames (ORFs) are shown as arrows in different colors. Direction of arrowhead represents transcriptional orientation. The inner circle shows a GC plot. (B) Partial sequence map of pVc1 containing the *pirAB* homologues, the *transposases* genes (*tnp*) (green) and the terminal inverted repeats (IRs).

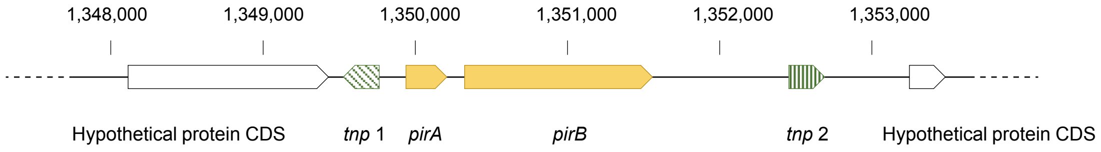

Fig. S5 Partial genomic sequence map of *Shewanella violacea*. The *pirAB* homologues are shown in yellow, and the two different transposase genes are shown in green.
